# 17q12 deletion syndrome presenting with chronic pancreatitis: a case report

**DOI:** 10.3389/fmed.2026.1775502

**Published:** 2026-04-15

**Authors:** Meng-zhen Liu, Xiao-fei Zhang, Yan Shao, Chun-di Guan, Shu-chen Dong, Wei Wang

**Affiliations:** 1 Binzhou Medical University Hospital, Binzhou, Shandong, China; 2 Shandong University of Aeronautics, Binzhou, Shandong, China

**Keywords:** 17q12 deletion syndrome, chronic pancreatitis, MODY5, MRKH syndrome, renal cysts

## Abstract

**Introduction:**

17q12 deletion syndrome is a rare autosomal dominant disorder classically characterized by renal cystic disease, maturity-onset diabetes of the young type 5 (MODY5), and Müllerian duct anomalies (e. g., MRKH syndrome).

**Case presentation:**

Pancreatic manifestations in this syndrome commonly include congenital structural abnormalities (e.g., dorsal agenesis) or atrophy, whereas classic chronic pancreatitis is rarely documented. We report an 18-year-old female with recurrent upper abdominal pain, steatorrhea, and dyspepsia. Imaging revealed pancreatic atrophy with calcifications. Whole-exome sequencing confirmed a diagnosis of 17q12 deletion syndrome.

**Conclusion:**

This case is the first to identify chronic pancreatitis as a significant clinical phenotype of 17q12 deletion syndrome. By integrating a literature review, we discuss the pathophysiology related to hepatocyte nuclear factor 1β (HNF1B) haploinsufficiency, suggesting that chronic pancreatitis may constitute part of the syndrome's clinical spectrum.

## Introduction

17q12 deletion syndrome is a rare genetic disorder caused by a heterozygous deletion in the chromosomal region 17q12, typically spanning 1.4 to 1.65 Mb and encompassing approximately 15 genes (1–3). Haploinsufficiency of the hepatocyte nuclear factor 1β (HNF1B) gene is considered pivotal for the renal and pancreatic manifestations (4, 5). The clinical presentation is heterogeneous. The classic triad comprises renal abnormalities (particularly cystic kidney disease), maturity-onset diabetes of the young type 5 (MODY5), and female Müllerian duct anomalies (MRKH syndrome), often accompanied by multisystem involvement such as neurodevelopmental disorders and liver dysfunction (6). Pancreatic involvement frequently includes endocrine dysfunction (MODY5) and structural anomalies (e.g., hypoplasia, atrophy, or cysts). However, cases exhibiting typical clinical and imaging features of chronic pancreatitis have not been systematically reported (7–11). This article explores the potential association between 17q12 deletion syndrome and chronic pancreatitis via a case of a young female patient with prominent chronic pancreatitis features.

## Case details

An 18-year-old female was admitted on December 14, 2024, with recurrent upper abdominal pain, steatorrhea, and dyspepsia. The patient reported intermittent epigastric pain for approximately 6 months prior to admission, which had gradually worsened in frequency and intensity. The patient had no prior episodes of recurrent acute pancreatitis and no history of smoking or alcohol consumption. Laboratory tests showed normal serum calcium and lipid levels, and immunological testing, including IgG4, was within the normal range (Table 1). These findings helped exclude common etiologies of chronic pancreatitis, including alcohol-related pancreatitis, metabolic causes, and autoimmune pancreatitis. Her medical history included congenital uterine agenesis and a 4-year history of diabetes mellitus, for which she was initially diagnosed with type 1 diabetes at age 14 and treated with insulin. Both parents developed diabetes after age 40, which was clinically classified as type 2 diabetes. At that time, the patient's extra-pancreatic manifestations were not yet recognized as part of a syndromic disorder, so monogenic diabetes was not initially suspected. Subsequent testing during this admission, however, revealed negative diabetes autoantibodies [including anti-glutamate decarboxylase antibodies (GAD) and insulin autoantibodies], which, in conjunction with her family history and syndromic features, argued against a diagnosis of classic type 1 diabetes (Table 1). Despite insulin therapy, her glycemic control remained suboptimal, with a most recent HbA1c level of 9.1%, indicating poor long-term glycemic control.

**Table 1:** Summary of laboratory data.

Parameter	Result	Unit	Reference range
GLU	16.78	mmol/l	3.9–6.1
K^+^	3.49	mmol/l	3.5–5.3
Ca^2+^	2.33	mmol/l	2.2–2.7
Mg^2+^	0.37	mmol/l	0.75–1.02
c-p	0.4	ng/ml	1.1–4.4
HbA1C	9.1	%	4.0–6.0
IgG4	0.011	g/L	≤ 2
GAD	1.02	IU/mL	0–10
IAA	0.03	Col	0–1.1
TG	1.24	mmol/l	0–1.7
TC	3.21	mmol/l	0–5.18

GLU, Glucose; c-p, C-peptide; HbA1C, Glycated hemoglobin; GAD, Anti-glutamate decarboxylase antibodies; IAA, Anti-insulin antibodies; TG, Triglycerides; TC, Total cholesterol.

Pancreatic endocrine and exocrine functions were assessed. A fasting C-peptide level of 0.40 ng/mL confirmed severely reduced pancreatic endocrine function, consistent with an insulin-deficient state such as MODY5. This was corroborated by persistent hyperglycemia on glycemic monitoring. Pancreatic exocrine insufficiency was suspected based on the presence of steatorrhea and dyspeptic symptoms. Although fecal elastase-1 testing was not performed, the diagnosis was supported by the combination of characteristic symptoms, imaging evidence of pancreatic atrophy, and a favorable clinical response to pancreatic enzyme replacement therapy.

**Figure 1:**
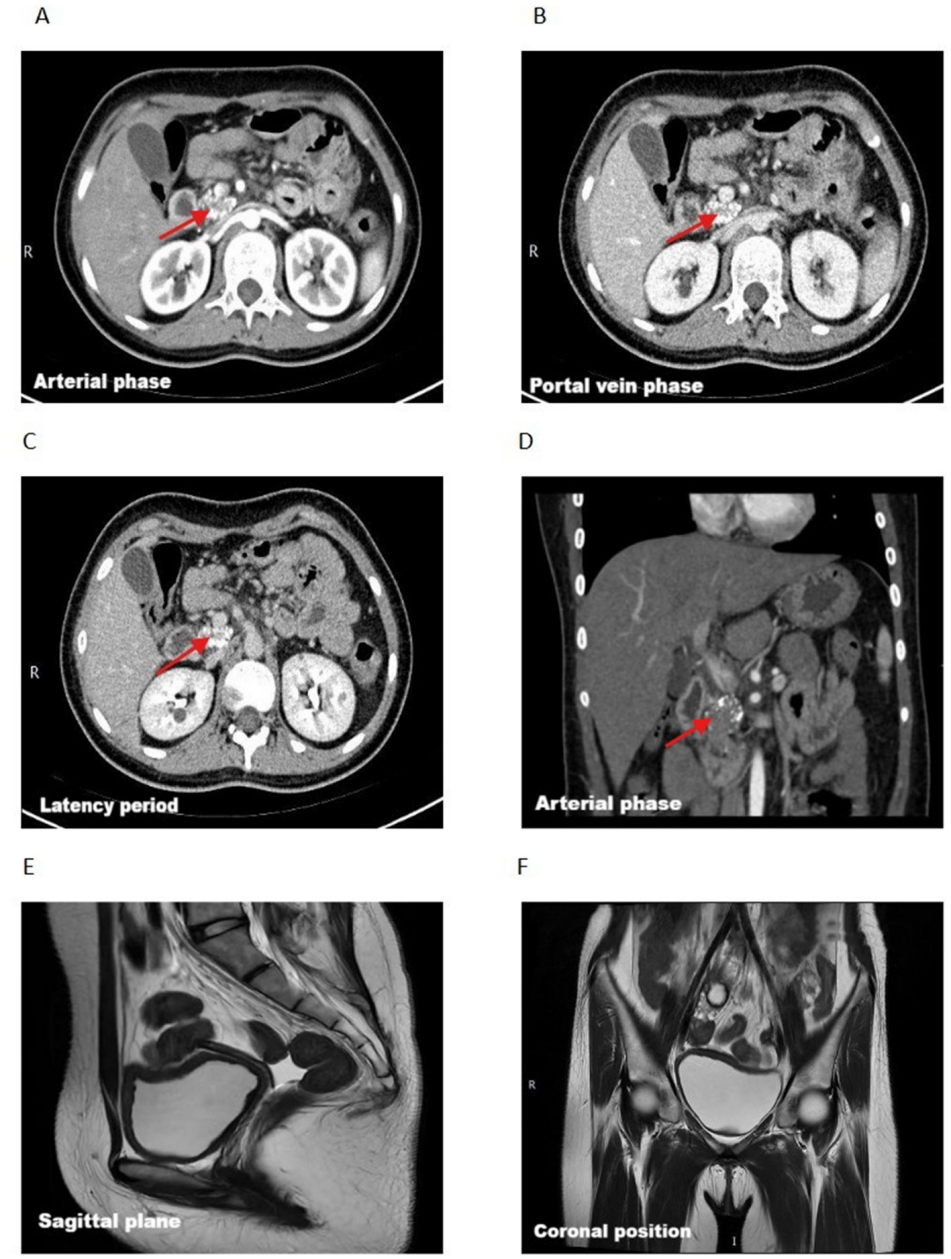
Bdominal contrast-enhanced CT and pelvic MRI findings. **(A–C)** Axial contrast-enhanced CT images obtained during the arterial phase (**A**), portal venous phase **(B)**, and delayed phase **(C)** demonstrate multiple patchy calcifications in the uncinate process of the pancreatic head (red arrows). **(C)** In the delayed phase, bilateral kidneys show multiple rounded hypodense non-enhancing lesions, consistent with renal cysts, with the largest measuring approximately 1.4 cm in diameter. **(D)** Coronal CT reconstruction shows marked pancreatic atrophy with non-visualization of the pancreatic body and tail (red arrow). **(E)** Pelvic MRI sagittal view shows absence of the uterus with poorly defined vaginal structures. **(F)** Pelvic MRI coronal view demonstrates bilateral ovaries located adjacent to the psoas major muscles.

**Figure 2:**
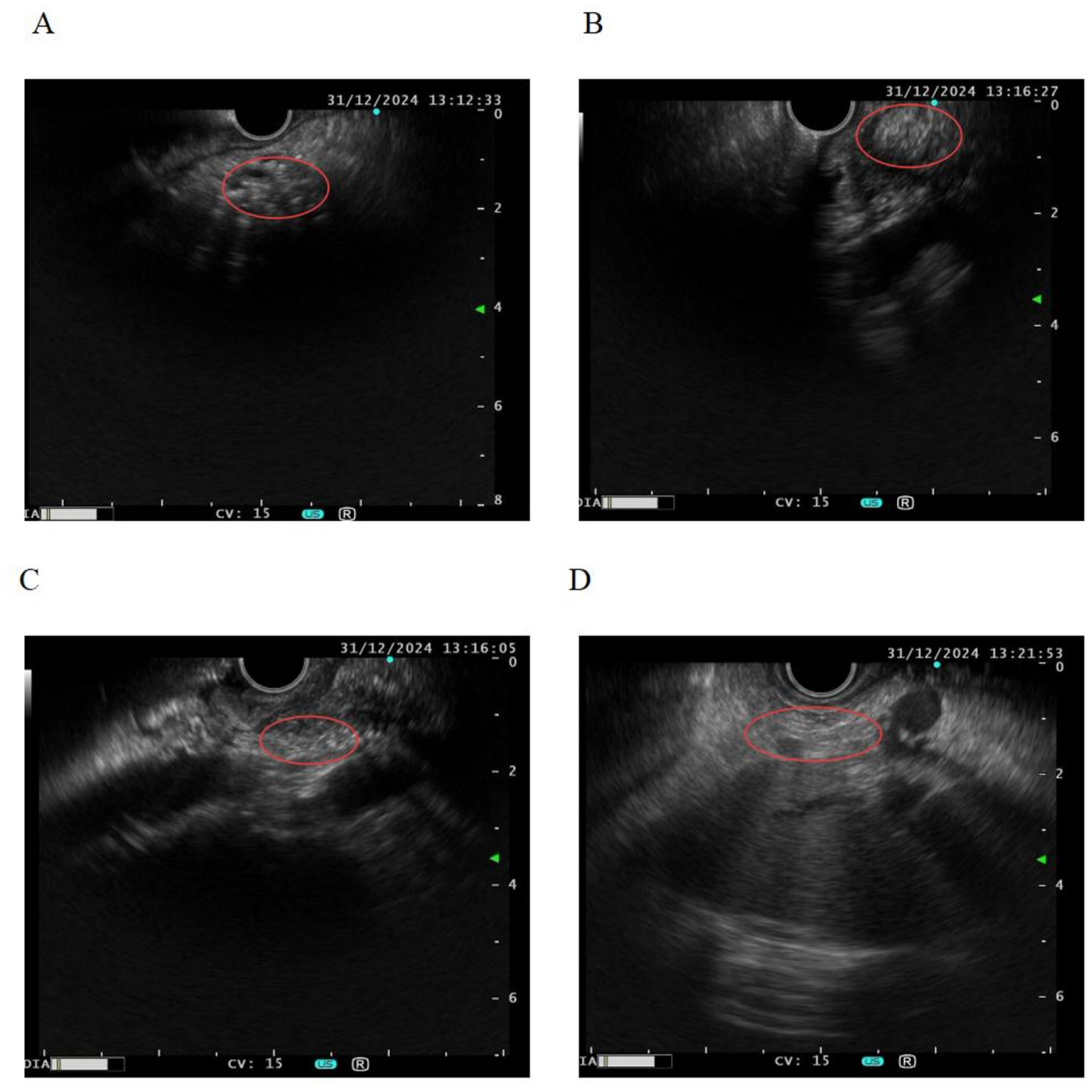
Endoscopic ultrasound. The volume of the pancreatic body and tail is significantly atrophic, with the parenchymal echo showing poor homogeneity. The head of the pancreas presents as short-stick, strand-like, and lobular hyper-echoic structures, with marked calcification and a slender pancreatic duct. **(A)** Head of the pancreas (atrophy and calcification in the pancreatic head region). **(B, C)** Calcification is present in the sulcal region of the pancreatic head. **(D)** Pancreatic body and tail (marked atrophy of the pancreatic body and tail).

Contrast-enhanced abdominal CT scan showed non-visualization of the pancreatic body and tail, multiple patchy calcifications in the uncinate process, and overall pancreatic atrophy. The kidneys demonstrated multiple non-enhancing rounded hypodense lesions (maximum diameter approximately 1.4 cm). Magnetic resonance cholangiopancreatography (MRCP) was not performed in this patient due to clinical and logistical constraints. Pelvic MRI confirmed uterine agenesis, ill-defined vaginal structure, and bilateral ovaries located adjacent to the psoas major muscles (Figure 1). Endoscopic ultrasound revealed heterogeneous echotexture in the pancreatic head with multiple ductal calcifications, consistent with chronic pancreatitis (Figure 2).

**Figure 3:**
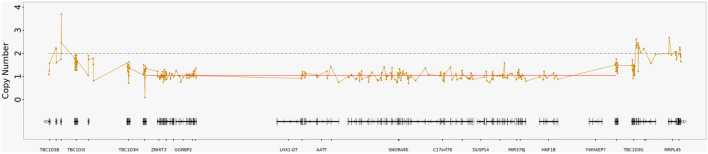
Genomic schematic of the 17q12 microdeletion identified in the patient. This figure illustrates a deletion in the chr17q12 region (GRCh37: 34842543–36104875), which corresponds to the 17q12 recurrent (RCAD syndrome) region (including HNF1B), a known haploinsufficiency region in the ClinGen database. The horizontal axis of the figure shows a schematic representation of the genes contained within the region, while the vertical axis represents copy number. The copy number for the deleted region is 1, and the copy number for the normal regions at either end is 2.

Whole-exome sequencing identified an approximately 1.26 Mb copy number deletion in the chr17q12 region (GRCh37: 34842543-36104875), encompassing genes including HNF1B, ACACA, MYO19, TADA2A, and ZNHIT3. Parental samples were unavailable for testing; therefore, the inheritance pattern could not be definitively determined. However, neither parent nor the patient's sibling had clinical features suggestive of 17q12 deletion syndrome, indicating that the deletion may represent a *de novo* event (Figure 3). In addition, no pathogenic variants were identified in known pancreatitis-associated genes, including PRSS1, SPINK1, CFTR, CTRC, CPA1, and TRPV6.

Based on these findings, a diagnosis of 17q12 deletion syndrome was established. The patient received comprehensive management, including pancreatic enzyme replacement, analgesia, insulin therapy for glycemic control, correction of ketosis and electrolyte imbalances, and nutritional support. Her symptoms improved significantly, with subsequent stabilization of blood glucose and electrolyte levels and gradual weight gain.

## Discussion

A search of the PubMed database up to October 2025 identified 58 reported cases of 17q12 deletion syndrome. Literature indicates that affected individuals often exhibit multi-system involvement, including renal anomalies (e.g., cysts, dysplasia), pancreatic structural abnormalities, MRKH syndrome, neurodevelopmental disorders (e.g., intellectual disability, autism spectrum disorder), and liver dysfunction (12, 13). We systematically reviewed 23 prior cases with pancreatic involvement. All exhibited varying degrees of pancreatic structural anomalies (e.g., hypoplasia, abnormal main pancreatic duct) and endocrine dysfunction (MODY5). The present case, displaying pancreatic body/tail agenesis and MODY5, aligns with these reports.

Notably, our patient also exhibited clear symptoms of exocrine pancreatic insufficiency, including abdominal pain, steatorrhea, and dyspepsia. Imaging demonstrated pancreatic atrophy with extensive calcifications, conforming to the classic presentation of chronic pancreatitis. Both contrast-enhanced CT and endoscopic ultrasound supported this diagnosis. The patient is a young female in whom common etiologies of chronic pancreatitis—such as alcoholic, biliary, and autoimmune causes—were excluded. Given her history of congenital uterine agenesis, a genetic etiology was suspected. Subsequent genetic testing confirmed 17q12 deletion syndrome.

The identified deletion contains several genes, including HNF1B, ACACA, MYO19, TADA2A, and ZNHIT3, meeting the molecular diagnostic criteria for 17q12 deletion syndrome. HNF1B is the key pathogenic gene. Its haploinsufficiency is closely associated with renal disease (e.g., renal cysts, refractory hypomagnesemia) and pancreatic abnormalities (MODY5, hypoplasia), often accompanied by systemic manifestations such as neurodevelopmental disorders (12, 14). This patient demonstrates typical HNF1B-related phenotypes, including bilateral renal cysts, hypomagnesemia, MODY5, and pancreatic body/tail agenesis, but lacks overt neurological or psychiatric symptoms. Furthermore, the patient has MRKH syndrome. Literature suggests that HNF1B and LHX1 genes cooperatively regulate Müllerian duct development, and their deletion can lead to uterine agenesis or vaginal atresia (15, 16). Genes such as ACACA, MYO19, TADA2A, and ZNHIT3 are also implicated in neurodevelopment, although our patient did not exhibit related symptoms, underscoring the phenotypic heterogeneity of this syndrome (7, 17).

HNF1B plays a crucial role in the differentiation, homeostasis, and function of pancreatic ductal cells. Although direct evidence linking HNF1B haploinsufficiency to human chronic pancreatitis is limited, animal studies offer relevant insights. Research using mouse models has demonstrated that specific knockout of HNF1B in pancreatic ductal cells can induce pathological changes highly reminiscent of human chronic pancreatitis (18). We therefore postulate that the 17q12 deletion in this patient, resulting in HNF1B haploinsufficiency, may impair not only embryonic pancreatic development but also postnatal ductal cell homeostasis and repair capacity (19). Under prolonged metabolic or other stresses, the pancreas may become susceptible to recurrent injury, initiating an “injury-inflammation-fibrosis” vicious cycle and ultimately progressing to classic chronic pancreatitis (18).

Previous reports have described pancreatic abnormalities associated with HNF1B deficiency, including pancreatic hypoplasia, dorsal pancreatic agenesis, and endocrine dysfunction. Notably, Motyka et al. (20) reported a patient with complete HNF1B gene deletion who presented with pancreatic calcifications and absence of the pancreatic tail. These findings suggest that HNF1B haploinsufficiency may predispose individuals to pancreatic structural abnormalities and calcification.

In the present case, the patient harbored a 1.26 Mb microdeletion in the 17q12 region, encompassing HNF1B and several adjacent genes. Although the genetic mechanism differs from an isolated HNF1B deletion, the resulting haploinsufficiency of HNF1B remains a shared pathogenic driver. Our findings therefore support the hypothesis that HNF1B-related disorders may predispose to chronic pancreatic injury and calcification, further expanding the pancreatic phenotype associated with 17q12 deletion syndrome.

Traditionally, pancreatic phenotypes in 17q12 deletion syndrome are categorized as developmental defects, with imaging typically revealing atrophy or agenesis of the pancreatic body and tail. Pancreatic endocrine dysfunction is common, with an incidence up to 76% (10). In our patient, however, the presence of extensive patchy pancreatic calcifications extends beyond simple developmental anomaly and represents characteristic imaging findings of chronic pancreatitis. While literature mentions symptoms akin to chronic pancreatitis secondary to pancreatic atrophy, such cases often lack definitive calcification evidence. This case provides direct imaging evidence, thereby establishing a link between 17q12 deletion syndrome and classical chronic pancreatitis.

Several limitations should be acknowledged. First, magnetic resonance cholangiopancreatography (MRCP) was not performed, which may have provided additional information regarding the pancreatic ductal anatomy and helped exclude anatomical variants such as pancreas divisum. However, the diagnosis of chronic pancreatitis in this case was supported by characteristic findings on contrast-enhanced CT and endoscopic ultrasound. Second, the 17q12 deletion was identified through a copy number variation prediction algorithm based on whole-exome sequencing data. Although this approach can reliably detect large deletions, confirmatory techniques such as multiplex ligation-dependent probe amplification (MLPA), array comparative genomic hybridization (array CGH), or optical genome mapping (OGM) were not performed due to technical limitations. Therefore, further validation using dedicated CNV detection methods would strengthen the molecular findings.

## Conclusion

To our knowledge, reports describing chronic pancreatitis with pancreatic calcifications in patients with 17q12 microdeletion syndrome remain extremely limited. This case expands the phenotypic spectrum of 17q12 deletion syndrome and highlights that classical features of chronic pancreatitis may occur in young patients with HNF1B haploinsufficiency. It enhances the understanding of the pancreatic involvement spectrum in this syndrome from both molecular and clinical perspectives. For young patients with chronic pancreatitis, particularly those with MODY5 or congenital reproductive system anomalies, 17q12 deletion syndrome should be considered in the differential diagnosis. Early genetic testing can facilitate accurate diagnosis and guide personalized management.

## Data Availability

The original contributions presented in the study are included in the article/supplementary material, further inquiries can be directed to the corresponding author.

## References

[1] Zhang S Ma Y Zang X Heng H Liu X Peng G . A case of 17q12 microdeletion syndrome in a mody5 type diabetes with hnf-1beta gene mutation accompanied. Appl Clin Genet. (2024) 17:125–30. doi: 10.2147/TACG.S465859 39050772 PMC11268705

[2] Roehlen N Hilger H Stock F Glaser B Guhl J Schmitt-Graeff A . 17q12 deletion syndrome as a rare cause for diabetes mellitus type mody5. J Clin Endocrinol Metab. (2018) 103:3601–10. doi: 10.1210/jc.2018-00955 30032214

[3] Luo X Chen X Cong X Niu H Zhou F Song J . Prenatal diagnosis, ultrasound findings, and pregnancy outcome of 17q12 deletion and duplication syndromes: a retrospective case series. Arch Gynecol Obstet. (2024) 310:2921–30. doi: 10.1007/s00404-024-07789-4 39433644

[4] Laffargue F Bourthoumieu S Llanas B Baudouin V Lahoche A Morin D . Towards a new point of view on the phenotype of patients with a 17q12 microdeletion syndrome. Arch Dis Child. (2015) 100:259–64. doi: 10.1136/archdischild-2014-306810 25324567

[5] Zhang F Gu Q Song J Zhao Y Wang Z Men S . Prenatal diagnosis and family analysis of 17q12 microdeletion syndrome with fetal renal abnormalities. Front Genet. (2024) 15:1401315. doi: 10.3389/fgene.2024.1401315 38957807 PMC11217314

[6] Lee R Choi JE Mun E Kim KH Choi SA Kim HS. A case of chromosome 17q12 deletion syndrome with type 2 mayer-rokitansky-kuster-hauser syndrome and maturity-onset diabetes of the young type 5. Children. (2024) 11:404. doi: 10.3390/children11040404 38671621 PMC11049139

[7] Gambella A Kalantari S Cadamuro M Quaglia M Delvecchio M Fabris L . The landscape of hnf1b deficiency: a syndrome not yet fully explored. Cells. (2023) 12:307. doi: 10.3390/cells12020307 36672242 PMC9856658

[8] Rico-Rodriguez M Fuentes-Cantero S Garcia-Rivera MC Varo-Sanchez GM. Pediatric patient with maturity-onset diabetes of the young type 5 and 17q12 deletion syndrome: a case report. Med Int. (2025) 5:61. doi: 10.3892/mi.2025.260 40901483 PMC12400136

[9] Aydin C Kiral E Susam E Tufan AK Yarar C Cetin N . A case of familial recurrent 17q12 microdeletion syndrome presenting with severe diabetic ketoacidosis. Turk J Pediatr. (2022) 64:558–65. doi: 10.24953/turkjped.2021.1613 35899569

[10] Clissold RL Fulford J Hudson M Shields BM McDonald TJ Ellard S . Exocrine pancreatic dysfunction is common in hepatocyte nuclear factor 1β-associated renal disease and can be symptomatic. Clin Kidney J. (2018) 11:453–8. doi: 10.1093/ckj/sfx150 30094008 PMC6070112

[11] Guzman GE Madariaga I Vargas CJ Galeano LB Guerra MA Nastasi JA. Identification of 17q12 microdeletion syndrome in a latin american patient with maturity-onset diabetes of the young subtype 5: a case report. J Med Case Rep. (2023) 17:152. doi: 10.1186/s13256-023-03873-6 37016461 PMC10074670

[12] Nittel CM Dobelke F Konig J Konrad M Becker K Kamp-Becker I . Review of neurodevelopmental disorders in patients with hnf1b gene variations. Front Pediatr. (2023) 11:1149875. doi: 10.3389/fped.2023.1149875 36969268 PMC10034397

[13] Tutulan-Cunita A Pavel AG Dimos L Nedelea M Ursuleanu A Neacsu AT . Phenotypic variability of 17q12 microdeletion syndrome - three cases and review of literature. Balkan J Med Genet. (2021) 24:71–82. doi: 10.2478/bjmg-2021-0025 36249519 PMC9524179

[14] Quintero-Rivera F Woo JS Bomberg EM Wallace WD Peredo J Dipple KM. Duodenal atresia in 17q12 microdeletion including hnf1b: a new associated malformation in this syndrome. Am J Med Genet A. (2014) 164A:3076–82. doi: 10.1002/ajmg.a.36767 25256560

[15] Thomson E Tran M Robevska G Ayers K van der Bergen J Gopalakrishnan Bhaskaran P . Functional genomics analysis identifies loss of hnf1b function as a cause of mayer-rokitansky-küster-hauser syndrome. Hum Mol Genet. (2023) 32:1032–47. doi: 10.1093/hmg/ddac262 36282544 PMC9990990

[16] Pontecorvi P Bernardini L Capalbo A Ceccarelli S Megiorni F Vescarelli E . Protein-protein interaction network analysis applied to dna copy number profiling suggests new perspectives on the aetiology of mayer-rokitansky-küster-hauser syndrome. Sci Rep. (2021) 11:448. doi: 10.1038/s41598-020-79827-5 33432050 PMC7801512

[17] Anttonen A Laari A Kousi M Yang YJ Jääskeläinen T Somer M . Znhit3 is defective in peho syndrome, a severe encephalopathy with cerebellar granule neuron loss. Brain J Neurol. (2017) 140:1267–79. doi: 10.1093/brain/awx040 28335020

[18] Quilichini E Fabre M Dirami T Stedman A De Vas M Ozguc O . Pancreatic ductal deletion of hnf1b disrupts exocrine homeostasis, leads to pancreatitis, and facilitates tumorigenesis. Cell Mol Gastroenterol Hepatol. (2019) 8:487–511. doi: 10.1016/j.jcmgh.2019.06.005 31229598 PMC6722301

[19] Omura Y Yagi K Honoki H Iwata M Enkaku A Takikawa A . Clinical manifestations of a sporadic maturity-onset diabetes of the young (mody) 5 with a whole deletion of hnf1b based on 17q12 microdeletion. Endocr J. (2019) 66:1113–6. doi: 10.1507/endocrj.EJ19-0020 31391355

[20] Motyka R Kołbuc M Wierzchołowski W Beck BB Towpik IE Zaniew M. Four cases of maturity onset diabetes of the young (mody) type 5 associated with mutations in the hepatocyte nuclear factor 1 beta (hnf1b) gene presenting in a 13-year-old boy and in adult men aged 33, 34, and 35 years in poland. Am J Case Rep. (2021) 22:e928994. doi: 10.12659/AJCR.928994 33526762 PMC7869582

